# A Comprehensive Review of COVID-19 Virology, Vaccines, Variants, and Therapeutics

**DOI:** 10.1007/s11596-021-2395-1

**Published:** 2021-07-09

**Authors:** Lauren Forchette, William Sebastian, Tuoen Liu

**Affiliations:** Department of Biomedical Sciences, West Virginia School of Osteopathic Medicine, 400 Lee Street North, Lewisburg, West Virginia 24901 USA

**Keywords:** severe acute respiratory syndrome coronavirus 2, coronavirus disease 2019, vaccines, variant strains, antiviral therapy

## Abstract

Severe acute respiratory syndrome coronavirus 2 (SARS-CoV-2), the causative pathogen of the coronavirus disease 2019 (COVID-19), has caused more than 179 million infections and 3.8 million deaths worldwide. Throughout the past year, multiple vaccines have already been developed and used, while some others are in the process of being developed. However, the emergence of new mutant strains of SARS-CoV-2 that have demonstrated immune-evading characteristics and an increase in infective capabilities leads to potential ineffectiveness of the vaccines against these variants. The purpose of this review article is to highlight the current understanding of the immunological mechanisms of the virus and vaccines, as well as to investigate some key variants and mutations of the virus driving the current pandemic and their impacts on current management guidelines. We also discussed new technologies being developed for the prevention, treatment, and detection of SARS-CoV-2. In this paper, we thoroughly reviewed and provided crucial information on SARS-CoV-2 virology, vaccines and drugs being used and developed for its prevention and treatment, as well as important variant strains. Our review paper will be beneficial to health care professionals and researchers so they can have a better understanding of the basic sciences, prevention, and clinical treatment of COVID-19 during the pandemic. This paper consists of the most updated information that has been available as of June 21, 2021.

### 1 INTRODUCTION AND VIROLOGY

Coronaviruses are a diverse group of enveloped, single-stranded, positive-sense RNA viruses that have a wide-ranged tropism, giving them the ability to cause devastating diseases. They have the largest genome of all RNA-based viruses with 26–32 kb, along with a 5′ and 3′ poly-A tail[1–3]. The three most notable coronaviruses that affect the human population are SARS-CoV-1 in 2002 and MERS-CoV in 2012, both of which caused epidemics, and SARS-CoV-2 in 2019, which is the causative agent of the current coronavirus disease 2019 (COVID-19) pandemic. According to the World Health Organization (WHO) Dashboard (https://covid19.who.int/), as of June 21, 2021, there have been 178 118 597 confirmed cases and 3 864 180 deaths of COVID-19 globally.

The key structural components of the pathologic strains include the membrane, envelope, nucleocapsid, and spike proteins, sharing the core conservative elements across the coronavirus family[4]. SARS-CoV-1 and SARS-CoV-2 share 94.6% amino acid sequence along with 80% nucleotide base pairing[5]. Although SARS-CoV-2 encodes the four core conservative elements, it is significantly different from previous pathologic coronavirus strains because it also expresses 8 accessory proteins [3a, 3b, p6, 7a, 7b, 8b, 9b, and open reading frame (ORF)14] which are not all conservative among SARS-CoV-2, SARS-CoV-1, and MERS-CoV[2].

The major entry receptor for both SARS-CoV-1 and SARS-CoV-2 binding is the angiotensin-converting enzyme 2 (ACE2) on host cells contrasting with MERS-CoV which utilizes dipeptidyl peptidase 4 or CD26 for entry. The SARS-CoV-2 binds with ACE2 via its spike protein (S protein). The S protein is composed of 2 subunits, S1 and S2, with each S protein consisting of three S1/S2 subunits that create a trimer (fig. 1A)[6, 7]. The S1 subunit is responsible for the binding of ACE2 via the receptor-binding domain (RBD), whereas S2 contains the fusion peptide as well as the transmembrane domain[7]. The entry of SARS-CoV-2 into host cells depends on two key

Lauren Forchette, E-mail: lforchette@osteo.wvsom.edu; William Sebastian, E-mail: wsebastian@osteo.wvsom.edu
[†]These authors contribute equally to this work.
[#]Corresponding author, E-mail: tliu@osteo.wvsom.edu

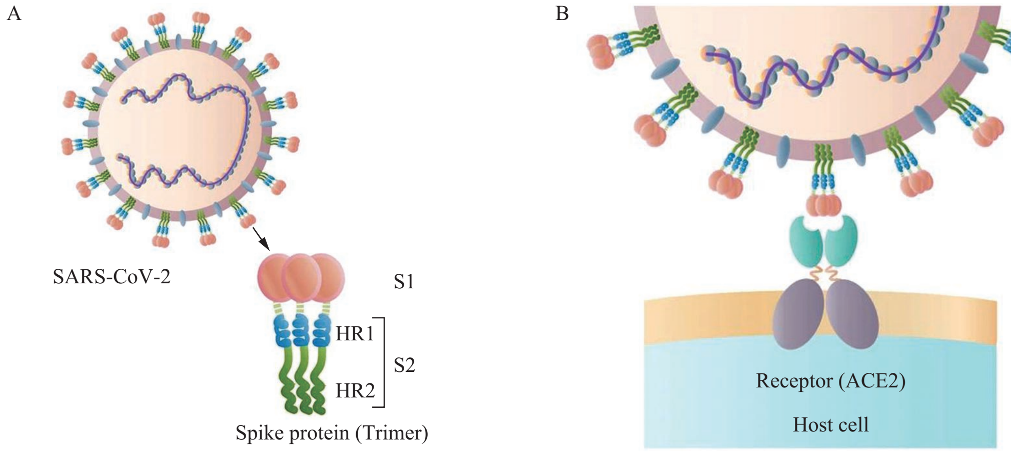

**Fig. 1** The schematic structure of SARS-COV-2
A: the structure of the S protein; B: the relationship between the viral S protein and host cell's ACE2 receptor factors: (1) binding of the viral spike proteins to host cellular receptor ACE2; and (2) S protein priming by the host cell protease, transmembrane protease serine 2 (TMPRSS2)[8–10]. ACE2 is a type Ⅰ transmembrane metallocarboxypeptidase with homology to ACE, presenting on multiple cell types such as endothelial cells, pneumocyte-2 in the lungs, and enterocytes. The utilization of ACE2 is a basis for many clinical symptoms presented in COVID-19 patients, and we will discuss in next paragraph. The RBD of the viral spike protein is significant for viral infectivity and the RBD between SARS-CoV-1 and SARS-CoV-2 shares 73%–76% conservation. It was found that the trimer formation of the S protein in SARS-CoV-2 had a higher affinity for the ACE2 receptor than SARS-CoV-1 (fig. 1B)[6, 7]. Mutations within the S protein can have a major impact on the tropism, infectivity, and ultimately the lethality of the virus.

Patients with COVID-19 present with diverse clinical presentations and symptoms including fatigue, fever, cough, loss of smell or tasting, and headache, etc. Around 30% of the patients also reported gastrointestinal symptoms such as diarrhea, nausea, and stomach pain[11, 12]. A study of 254 patients showed that the most common complication was pneumonia (82.3%), followed by arrhythmia (0.06%), and shock (0.03%)[11]. In patients hospitalized for COVID-19 infections, the most prevalent underlying conditions included age, obesity, hypertension, diabetes mellitus, heart disease, and lung disease[13, 14]. The way in which SARS-CoV-2 enters host cells can explain the wide variety of symptoms mentioned above, especially the function and locations of ACE2 receptors in viral entry. Specifically, viruses are first recognized by toll-like receptors on host cells, which initiate the activation of NF-κB, which then activates the ACE2 receptors on host endothelial cells in the respiratory tract, gastrointestinal tract, and kidney. Following the activation of ACE2 receptors, the viruses can then enter cells and begin replication[15]. This process also initiates the "cytokine storm", a hallmark of acute respiratory distress syndrome (ARDS) which is the leading cause of death due to severe infection of SARS-CoV-2[16, 17]. Patients with COVID-19 also have an increased risk of vascular crisis, which can be explained by the binding of S protein and ACE2. ACE2 converts angiotensin Ⅱ to angiotensin[1–7], protecting endothelial cell function and preventing atherosclerosis which can be caused by blood clots. When the ACE2 is bound to the viral S protein, the protective function of ACE2 is inhibited, leading to vascular occlusion in COVID-19 patients[18].

Upon entering the host cells, the single strand RNA genome can trigger an immune response via pathogen-associated molecular patterns (PAMPs)[19]. SARS-CoV-2 is a cytopathic virus which can cause pyroptosis (a highly inflammatory form of programmed death) of the host cells. The virus also causes the release of pro-inflammatory damage-associates molecular patterns (DAMPs) such as ATP and nucleic acids, as well as induces a local immune response with cytokines like IL-6. The DAMPs trigger the migration of other immune cells such as macrophages, monocytes, and T cells, which further increase the inflammation by releasing pro-inflammatory cytokines. In a susceptible immune system, the increase in viral load and inflammation lead to the cytokine storm, with severe cases showing an increase in IL-2, IL-7, IL-10, G-CSF, TNF-α, and macrophage inflammatory protein 1α (MIP1α), ultimately leading to multi-organ damage as well as septic shock[20].

In addition to the cytokine storm, T cells and B cells also play important roles in disease severity and defense. Zheng *et al* found that T cell exhaustion was associated with cytotoxicity reduction. They also reported that increased T cell exhaustion and reduced T cell function predicted severe diseases, as functional interferon-γ (IFN-γ) and tumor necrosis factor-α (TNF-α) of $CD4^+$ T cells were lower, and levels of

CD8+ perforin and granzyme B were higher in severe cases[21]. A significant function of the B cells against SARS-CoV-2 is neutralization. Neutralizing antibodies bind to the virus and block infection through two possible mechanisms: (1) by targeting the S protein and preventing its interaction with ACE2 receptors, or (2) by binding to a viral capsid in a manner that prevents uncoating of the genome[20]. A proposed adaptive immune response against SARS-CoV-2 which requires the stimulation of B and T cells is shown in fig. 2A[22]. Despite the need for appropriate B cell response to eradicate viral infection, a major flaw that counteracts the antibody effects is antibody-dependent enhancement (ADE), a phenomenon in which virus-specific antibodies enhance the entry and replication of virus into monocytes/macrophages and granulocytic cells through interaction with Fc and/or complement receptors[23, 24]. It is not fully understood how SARS-CoV-2 can exactly utilize this mechanism to exacerbate COVID-19, but proposed theories suggest that ADE may induce the severe symptoms seen, and explain why non-neutralizing antibodies

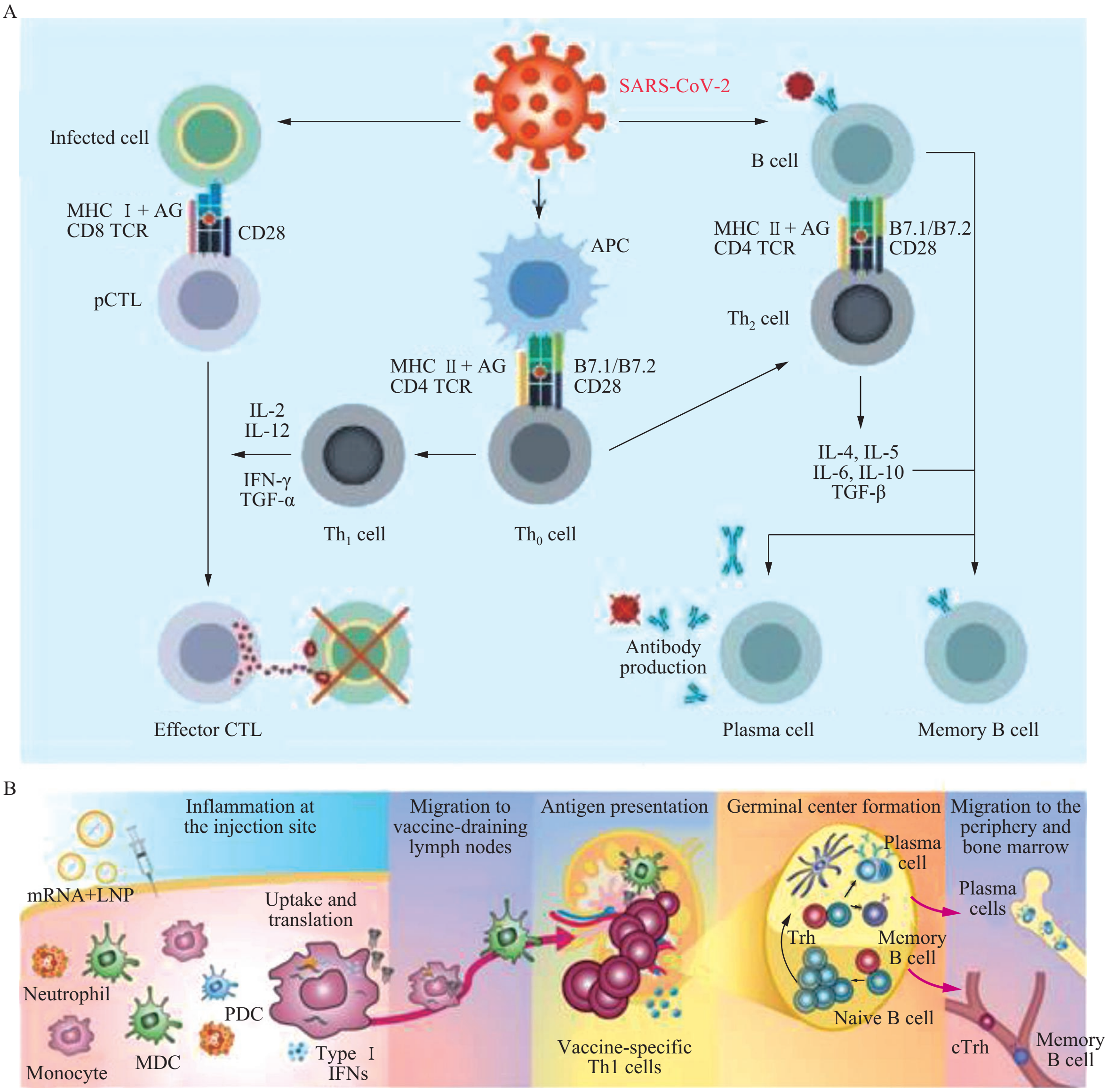

**Fig. 2** Immune response against SARS-CoV-2 infection and mRNA vaccine

A: The proposed adaptive immune response against coronavirus SARS-CoV-2 requires stimulation of B cell and T cell epitopes. After human cells are infected by virus entities, epitopes from viruses' proteins can be bound and presented by MHC-1 receptors on host cell surfaces, leading to the stimulation of CD4 and CD8 T cells to provoke antibody-mediated and cell-mediated immune responses; B: the proposed adaptive immune responses after mRNA vaccination. Local inflammation at the injection site promotes the infiltration of immune cells, including neutrophils, monocytes, myeloid dendritic cells (MDCs) and plasmacytoid dendritic cells (PDCs). Neutrophils can efficiently take up lipid nanoparticles, and monocytes and MDCs translate mRNA efficiently. Secretion of type Ⅰ interferons (IFNs) is stimulated. mRNA and protein antigen will disseminate and cells will migrate to the vaccine-draining lymph nodes. Antigen presentation to T cells and interactions of antigen and B cells take place at these sites, leading to the formation of germinal centers, which results in the generation of memory B cells and antibody-producing plasma cells that reside to the bone marrow.

produced by B cells enhance SARS-CoV-2 infection in some cases[25]. The ADE is also a consideration during vaccine development, and will be discussed in the "vaccine development and mechanisms" section. Many other variables, such as genetics, comorbidities, and health status, also contribute to the efficiency of an individual's immune response. A healthy innate defense can eradicate the pathogen, however, when the innate response is compromised, this can lead to increased viral load and tissue damage, and ultimately severe symptoms.

### 2 VACCINE DEVELOPMENT AND MECHANISMS

Throughout the past year, multiple vaccines against SARS-CoV-2 have either already been developed or are in the process of being developed worldwide. According to the WHO Dashboard, as of June 21, 2021, 2 412 226 768 vaccine doses have been administered globally. However, despite the rapid production and distribution of vaccines, several new strains originating from various regions globally are continuing to be discovered, and the current vaccines may not be sufficient to protect the population. Therefore, it is vital to understand the mechanism of current vaccines being produced, as well as investigate new research for potential new vaccines and treatments to prevent another wave of the current global pandemic.

Currently, there are three common types of vaccines against SARS-CoV-2 virus: mRNA, adenovirus vector, and inactivated vaccines. The mRNA vaccine generally consists only of the genetic code for a single antigen of the SARS-CoV-2 (e.g., S protein antigen), wrapped in a shell and delivered by lipid nanoparticles. When the mRNA vaccine is injected into the body, human cells use the viral genetic code to make the encoded antigen, then the body reacts and makes antibodies[26]. Unlike the mRNA vaccines, adenovirus vector vaccines generally use the DNA of the S protein antigen. Instead of using lipid nanoparticles to deliver the genetic code into the body, viral vector vaccines use a modified virus (different from SARS-CoV-2, such as adenovirus) as a vector to deliver the genetic information. When these adenoviruses containing DNA for the S protein enter the body, the viral proteins will be produced and further activates immune responses[27]. Inactivated vaccines use inactivated SARS-CoV-2 viruses, and viral inactivation is achieved by using chemicals, heat, or radiation. The inactivated viruses contain antigens, and when they are injected into the body, these antigens will provoke an immune response[28–30]. The major current vaccines being used and developed are listed in table 1.

The two currently well-known vaccines on the market, developed by Pfizer-BioNTech, Inc. (USA) and Moderna, Inc. (USA), are mRNA vaccines. The Pfizer-BioNTech vaccine was approved by the United States (U.S.) FDA to use in individuals of 16 years and older on December 11, 2020, and are currently authorized to use in more than 80 counties including USA, UK, and countries in the European Union. On May 10, 2021, the U.S. FDA expanded the emergency use authorization (EUA) to include adolescents of 12–15 years of age. The Moderna vaccine is authorized by U.S. FDA for use under an EUA for active immunization to prevent COVID-19 in individuals of 18 years of age and older on December 18, 2020. It is currently authorized for use in more than 40 countries including USA, Canada, UK, and European Union.

The mRNA vaccine developed by Moderna encodes the entire 1273 amino acid sequence of the viral S protein and is therefore named the mRNA-1273 vaccine. Pfizer-BioNTech, however, developed two vaccines: BNT162b1 (consisting of the RBD, a part of the S1 subunit of S protein) and BNT162b2 (consisting full-length of S-protein amino acid sequence, similar to the Moderna vaccine)[26]. A functional mRNA vaccine requires essential elements including the 5′ cap, 5′ UTR, ORF, 3′ UTR, and poly-A tail. When developing vaccines, the 5′ cap is often modified to ensure that no reverse binding occurs, which can decrease the efficiency of translation once the vaccine enters the host. It is also important to modify the 5′ and 3′ UTRs to optimize the mRNA sequence stability, along with the poly-A tail. The codons of the ORF are also optimized for translation activity and protein quality[31]. An advantage of using mRNA vaccines is that they can be modified by altering the sequence, which will be beneficial to make vaccines protective against new viral strains[31].

The robust host immune response after vaccine administration can be partly attributed to the self-adjuvant property of mRNA vaccines. After initial inflammation at the site of injection, the mRNA is translated and taken to lymph nodes, where it is recognized by antigen-presenting cells. Host toll-like receptors are then activated, which leads to cytokine production. The overarching result is a T cell response with elevated $CD8^+$ and Th1 type $CD4^+$ cells. $CD4^+$ T cells are crucial for B cell differentiation, which is necessary for the production of memory immune cells to protect host cells from future viral invasion. The proposed process of generation of adaptive immune responses by mRNA vaccination is illustrated in fig. 2B[32]. Currently, all available mRNA COVID-19 vaccines require a lipid nanoparticle component for delivery. Lipid nanoparticles assist the virus to penetrate host cell membrane. The schematic structure and functions of mRNA-lipid nanoparticle complex are illustrated in fig. 3[26, 33]. One disadvantage of using lipid nanoparticles as a delivery system is that they require

**Table 1 Vaccines in use and being developed**

| Country | Manufactory | Vaccine name | Approval status# | Mechanism | Route of administration | Efficacy | Side effects |
|---|---|---|---|---|---|---|---|
| USA | Pfizer-BioNTech | BNT162b2 & BNT162b1 | Approved | mRNA | IM | 94% | General* |
| USA | Moderna | mRNA-1273 | Approved | mRNA | IM | 94% | Similar to Pfizer-BioNTech |
| USA | Johnson & Johnson | Ad26.COV2.S | Approved | Viral vector | IM | 66% | Similar to Pfizer-BioNTech, but also causing thrombosis with thrombocytopenia syndrome |
| USA and Europe | AstraZeneca and Oxford University | AZD1222 | Approved (Phase Ⅲ clinical trial in USA) | Viral vector | IM | 70% | General, diarrhea, blood clots with low blood platelets, enlarged lymph nodes |
| USA | NovaVax | NVX-CoV2373 | Phase Ⅲ clinical trial | Recombinant nanoparticle | IM | 89.3% | Similar to Pfizer-BioNTech |
| China | Sinopharm | BBIBP-CoV | Approved | Inactivated virus | IM | 78% | General, scleroma, cough, difficulty in breathing |
| China | Sinovac | CoronaVac | Approved | Inactivated virus | IM | 50%–84% | No critical side effects |
| China | CanSinoBIO | Ad5-nCoV | Approved | Viral vector | IM | 67.2% | General, inappetence, constipation, mucocutaneous abnormalities |
| China | Wuhan Institute of Biological Products Co., Ltd. | VIVO4 | Phase Ⅲ clinical trial | Inactivated virus | IM | 72.8% | Serious side effects, rare |
| China | Beijing Institute of Biological Products Co., Ltd. | HBO2 | Phase Ⅲ clinical trial | Inactivated virus | IM | 78.1% | Serious side effects, rare |
| Russia | Gamaleya | Gam-COVID-Vac (or Sputnik V) | Approved | Viral vector | IM | 91.6% after 2 doses, 79.4% after 1 dose | No unusual side effects |
| UK | Iso-Bio | OraPro-COVID-19 | Clinical trial | Viral vector | Oral | Not reported | Not reported |
| USA | Vaxart | VXA-CoV2-1 | Phase Ⅰ clinical trial | Viral vector | Oral | Not reported | Not reported |
| Canada | Medicago | VIR-7831 | Phase Ⅲ clinical trial | Plant-based viral vector | IM | Not reported | Not reported |

##Approval status means the vaccines were approved by WHO, or U.S. FDA, or in certain countries. Currently, only three vaccines are approved by the USA: Pfizer-BioNTech, Moderna, and Johnson & Johnson. *General side effects including local effects (such as pain, redness, swelling at the injection sites) and systemic effects (such as tiredness, nausea, headache, muscle pain, joint pain, chills, fever, itching, and rash). IM: intramuscular injection

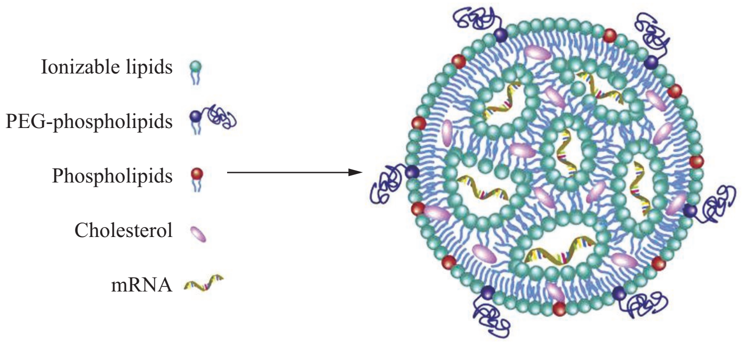

**Fig. 3** Schematic structure of mRNA-lipid nanoparticle complex

The lipid nanoparticles are used in mRNA delivery. The negative charge of mRNA electrostatically repulses the anionic (negatively charged) cell member, preventing its uptake into the cells. Therefore, mRNA vaccines require a delivery vehicle and the lipid nanoparticles are commonly used as mRNA carriers. Lipid nanoparticles are mainly composed of ionizable lipids, cholesterol, phospholipids, and polyethylene glycol (PEG)-lipid. The ionizable lipids are cationic (positively charged) at a low pH (enabling negatively-charged RNA complexation) and neutral at physiological pH (reducing potential toxic effects), allowing a better delivery of mRNA into the cells via endocytosis. Phospholipids play a structural role and cholesterol serves as a stabilizing element in lipid nanoparticles. Lipid-anchored PEGs dominantly deposit on the lipid nanoparticle surface as a barrier to sterically stabilize them and reduce nonspecific binding to proteins. Thus, the lipid nanoparticles have an efficient and safe profile for mRNA vaccine delivery.

the vaccines to be stored at frozen temperatures (–20°C or less) for long-term storage. If stored at refrigerated temperatures (2–8°C), the vaccines will only be stable for a short amount of time, approximately 5–30 days[34].

Different from Pfizer-BioNTech and Moderna, the manufactory Johnson & Johnson developed the vaccine Ad26.COV2.S (Research name: JNJ-78436735), which is an adenovirus vector vaccine, or viral vector vaccine. Although this vaccine still utilizes the S protein amino acid sequence of SARS-CoV-2, it requires adenovirus 26 DNA as a vector. The host immune response to the vaccine is still against SARS-CoV-2, and the immunologic mechanism by which it protects the host is similar to the mRNA vaccine as has described above[27]. One of the advantages of using a viral vector vaccine is the ability to rapidly produce the vaccines once the viral vector is synthesized in the lab. The Johnson & Johnson vaccine was approved by U.S. FDA under EUA for its single-dose vaccine to prevent COVID-19 in individuals of 18 years of age and older on February 27, 2021. The administration of the Johnson & Johnson viral vector vaccine was temporarily paused in mid-April 2021 due to its potential to cause cerebral venous sinus thrombosis, but resumed distribution in late April 2021 (benefits outweighed the risks) in the U.S.

The other vaccine (AZA1222), developed by AstraZeneca and Oxford University, is a viral vector vaccine. It was first approved in the United Kingdom (UK) on December 30, 2020, and has since been approved in multiple countries in Europe and Asia. However, it has not been approved by the U.S. FDA thus far. AZA1222 was suspended in some countries (e.g. South Africa, Europe, Canada) in February 2021, for similar reasons that the Johnson and Johnson vaccine was paused (mainly due to formation of blood clots)[35].

Currently, in addition to the U.S. and Europe, multiple COVID-19 vaccines have been approved and distributed to use globally, and several vaccine candidates are still in the clinical trials or are in the process of being approved. For example, China's Sinopharm BBIBP-CoV vaccine was approved for emergency use by the WHO on May 7, 2021. This vaccine is different from the vaccines previously approved for emergency use because it is an inactivated vaccine. This vaccine has similar effectiveness to the Johnson & Johnson and AstraZeneca vaccines at 78% efficacy and requires two doses intramuscularly (IM). BBIBP-CorV is being used in certain countries in Asia, Africa, South America, and Europe[29, 36]. Sinovac's CoronaVac is another inactivated vaccine being developed in China that also requires two doses IM. CoronaVac was approved in China and some other countries such as the Philippines and Cambodia in February 2021, and WHO validated the vaccine for emergency use on June 1, 2021. It showed 50%–84% of efficacy preventing COVID-19, and is being used in various countries in Asia, South America, North America, and Europe[30, 37]. China is also in the process of developing another viral vector vaccine (Ad5-nCOV, trade named: Convidecia), produced by CanSino Biologics. This vaccine uses adenovirus type 5 as a viral vector and was proven to be 67.2% effective in its phase Ⅲ clinical trial. It is authorized for use in China, Mexico, Pakistan, Hungary, Chile, Argentina and some other countries[29, 38–40]. A very recent phase Ⅲ clinical trial of two inactivated vaccines developed by China, WIVO04 and HB02, showed that they both significantly reduce the risk of symptomatic COVID-19 (the efficacy is 72.8% for WIVO4 and 78.1% for HBO2) and serious adverse events were rare[27].

Russia also developed an adenovirus viral vector vaccine, called Gam-Covid-Vac or Sputnik V, by the Gamaleya Research Institute of Epidemiology and Microbiology. It was registered on August 11, 2020 by the Russian Ministry of Health, and emergency mass-destitution began in December 2020 in countries including Russia, Argentina, Belarus, Hungary, Serbia and the United Arab Emirates. Although the vaccine requires 2 doses to reach its efficacy of 91.6%, Russia approved of giving the vaccine as a one-dose emergency administration, which had an efficacy of 79.4%[41, 42].

The Novavax COVID-19 vaccine, also known as NVX-CoV2373, is developed by Novavax and the Coalition for Epidemic Preparedness Innovations (CEPI). It is currently in phase Ⅲ clinical trial and was proven to be 89.3% effective. It requires two doses and is stable at 2 to 8°C. This vaccine is described as both a protein subunit vaccine and a virus-like particle vaccine, though the producers call it a "recombinant nanoparticle vaccine". The vaccine is produced by creating an engineered baculovirus containing a gene for a modified SARS-CoV-2 S protein[43–45].

While the majority of the vaccines being studied for protection against SARS-CoV-2 are to be injected IM, there have been a few companies investigating potential mucosal and oral delivery. Ios-Bio, a company based out of the UK, is in the process of developing a potential oral vaccine, OraPro-COVID-19. This vaccine encodes the S protein and requires a replication-defective Ad5 vector, similar to the vector used in CanSino's IM vaccine mentioned above. One of the potential benefits of oral vaccine delivery over IM is the storage ease. Comparing to intramuscular vaccines which require nanoparticles for delivery and cold temperature for storage, oral vaccines are delivered in a thermally stable capsule to avoid degradation in the gastrointestinal system, therefore, they do not need refrigeration for storage[46].

VXA-CoV2-1 is a non-replicating Ad5 vector adjuvanted oral tableted vaccine being developed by

Vaxart Inc. A phase Ⅱ trial will be started by mid-year in 2021. This vaccine will trigger host mucosal immunity by targeting the viral nucleocapsid (N) protein (a viral protein packaging the genome) as well as the S protein[47, 48]. This will be extremely advantageous given that the N protein is associated with the viral DNA and RNA and is less susceptible to mutations, meaning that this vaccine has the potential to more effective against the new variants due to the fact that most of the mutations in the new variants involve the S protein rather than the N protein.

Medicago, a company based in Quebec City, Canada, is attempting to produce a non-infectious, plant-based vaccine against COVID-19. The company had already been successful in producing a plant-based vaccine against influenza, and claimed one advantage of this vaccine, accuracy, in targeting specific strains of influenza. These plant-based vaccines utilize virus-like particles (VLPs) of SARS-CoV-2 (e.g., consisting of viral S protein). VLPs mimic the structure and function of the virus, but lack the genetic material required to infect host cells[49]. These VLPs are inserted into the bacterium Agrobacterium, which is taken up by plants in soil. Eventually, the plants are able to produce non-infectious and non-replicating VLPs in high volume, present a shell structure covered with the antigen required to trigger immune responses[50]. By mimicking the native structure of viruses, VLPs can be better recognized by the immune system because of their size, structure, and repeated antigen patterns are able to stimulate a more efficient immune response, compared with other types of vaccines. Moreover, this vaccine could have two additional advantages over the others: (1) they are cheaper to produce and will be beneficial in developing countries; and (2) they can precisely and rapidly protect against new mutant viral strains.

As discussed above, multiple countries and manufactures are in the process of developing COVID-19 vaccines, and some of them have been approved and used, with highly effective efficacy. A determining factor for the response to SARS-CoV-2 infection is the concentration of neutralizing antibodies found in the serum. Neutralizing antibodies might block interactions with receptors or bind to a viral capsid in a manner that prevents uncoating of the genome, further blocking infection. A recent study compared the post-vaccination concentration of neutralizing antibodies induced by major developed vaccines. It was found that mRNA-1273 and NVX-CoV23373 had the highest titer levels of neutralizing antibodies, and Ad26.COV2.S and CoronaVac had the lowest levels, indicating that Moderna and Novavax vaccines have the best outcome[51]. The study also indicated the concern about the length of immunity received from vaccinations because there was a significant loss to the levels of neutralizing antibodies 250 days post-vaccination. For example, a 95% efficacy vaccine would drop to 77%, whereas an initial efficacy of 70% would drop down to 33%. In addition to time after vaccination, variants of the SARS-CoV-2 strain also decreased antibody neutralization[51].

All vaccines are known to have certain side effects that consumers should be aware of before receiving their doses. Most of the vaccines are associated with local or injection and systemic side effects. Local reactions include pain, swelling, and erythema at the injection sites. Systemic effects, such as headaches, myalgia, fever, chills, and nausea, are mainly due to the body's immune response. The specific side effects of vaccines are listed in table 1.

As mentioned in the previous section, anti-SARS-CoV-2 antibodies could exacerbate COVID-19 through ADE mechanism. Previous respiratory syncytial virus and dengue virus vaccine studies revealed human clinical safety risks related to ADE, resulting in failed vaccine trials. Evidence from animal studies showed safety concerns of ADE for SARS-CoV-2 vaccine development and antibody-based therapy. SARS-CoV-2 antibodies bound to Fc receptors on macrophages and mast cells may represent two different mechanisms for ADE in patients, however, clinical data have not yet fully established a role for ADE in human COVID-19 pathology. In order to reduce the risks of ADE from immunotherapies, induction or delivery of high doses of potent neutralizing antibodies is preferred, as lower concentrations of non-neutralizing antibodies would be more likely to cause ADE[23, 25].

### 3 STRAINS OF CONCERN

The rush to develop and administer vaccines started as a means to protect the public from the only known strain of SARS-CoV-2 that was ravaging the population at the time, especially in highly populated areas. In the time since vaccines were developed in 2021, numerous new strains of SARS-CoV-2 have emerged and spiked public health concerns. On May 31, 2021, WHO announces simple, easy-to-say labels for SARS-CoV-2 variants of interest and concern, using letters of the Greek alphabet (e.g., Alpha, Beta, Gamma, and Delta). These new strains include but not limited to D614G, B.1.1.7 (also known as Alpha, VOC-202012/01, or 201/501Y.V1), B.1.526, B.1.351 (also known as Beta, or 501Y.V2), B.1.1.28.1 (including P1, also known as Gamma), and B.1.617. The common variant strains of SARS-CoV-2 are listed in table 2. These strains contain various mutations, and many of which are localized to the S protein that has resulted in changes in viral behavior and pathogenesis such as alterations to RBD and escaping from natural immunity[52]. The emergence of variant strains is undoubtedly a great threat to the control of the COVID-19 pandemic[53]. There is still

**Table 2 Common variant strains of SARS-CoV-2**

| New WHO name | Strain name | Other name(s) | Major mutation sites | First reported place | First reported time | Therapeutics |
|---|---|---|---|---|---|---|
| | D614G | | D614G | German, China | January 2020 | Pfizer-BioNTech vaccine showed decreased neutralization; Moderna no changes. |
| Alpha | B.1.1.7 | VOC-202012/01, or 201/501Y.V1 | D614G, a two amino acid deletion at positions 69-70, N501Y, P681H | UK | December 2020 | Moderna and Novavax vaccines showed decreased neutralization. |
| | B.1.526 | | E484K, S447N, L5F, T95I, D253G, D614G, A701V | New York, USA | November 2020 | Pfizer-BioNTech and Moderna vaccines showed no change in S477N, but decreased neutralization on E484K. |
| Beta | B.1.351 | 501Y.V2 | K417N, E484K, N501Y | South Africa | Late 2020 | NVX-CoV2373 vaccine showed good neutralization; Moderna vaccine showed decreased neutralization. |
| | B.1.1.28.1 | | E484K | Brazil | February 2020 | Covaxin vaccine showed good neutralization; Pfizer-BioNtech and Moderna vaccines showed decreased neutralization. |
| Gamma | P1 | 20J/501Y.V3, belongs to B.1.1.28 strain lineage | K417T, E484K, N501Y | Brazil, Japan | Early 2021 | mAb effects vary; Pfizer-BioNtech and Moderna vaccines showed decreased neutralization. |
| Delta | B.1.617 | | D111D, G142D, L452R, E484R, E484Q, D614G, P681R | India | Late 2020 | Pfizer-BioNtech and Moderna vaccines showed decreased neutralization. |

much concern about the emerging and surrounding new viral strains of the virus, which are causing pandemic in particular countries and regions such as UK, South Africa, and India[54]. We will discuss the virology and therapeutics of common variants.

One of the first major mutations to the original SARS-CoV-2 viral genome sequence, a point mutation of D614G in the S protein that resulted in the G614 strain, occurred early in the pandemic. This strain was originally found in Germany and China in late January 2020 and eventually spread globally. The change in genome seemed to always be accompanied by three other mutations: a C-to-T mutation in the 5′ UTR at position 241, an analogous mutation of C-to-T mutation at position 3037, and a C-to-T mutation at position 14 408 in the RNA-dependent RNA polymerase (RdRp) gene[55, 56]. The mutation was found to both increase infectivity and viral replication within human tissue compared to the original D614 virus, although the increase in infectivity did not increase in lethality[8, 55, 57]. The higher infectivity is associated with an increase in affinity of the RBD from the acquired mutation, as glycine (G) instead of aspartic acid (D) residue could allow for an increase in flexibility to the trimeric S protein structure and better affinity[8]. Regarding the therapeutic effects on the mutant strain, studies showed that the Pfizer-BioNTech BNT162b2 vaccine which was based on the original D614 sequence had a 1.7–2.0 decrease in neutralization, making it less effective[55, 57]. The Moderna mRNA-1273 vaccine showed similar neutralization when compared to the original strain[58].

The pathologic B.1.1.7 (also known as Alpha, VOC-202012/01, or 201/501Y.V1) was first noted in the UK in December, 2020, but currently being detected in over 40 countries. This strain has 17 non-synonymous mutations, along with 8 S protein mutations and the D614G mutation. Three out of the 8 S protein mutations are notable including a two-amino acid deletion at positions 69–70, N501Y, and P681H[59]. N501Y has been shown to increase the RBD affinity to ACE2, similar to the D614G mutation. The Moderna and Novavax vaccines exhibited only a moderate reduction in neutralization of the B1.1.7 strain *in vitro* compared to the original strain[59, 60].

B.1.526 strain was first identified in New York City in the U.S. in November 2020. Since its identification, the strain's prevalence has increased exponentially in the state of New York and its surrounding areas. Mutations in the B.1.526 strain include E484K (the most notable) and S447N in the S protein along with 5 common others including L5F, T95I, D253G, D614G, and A701V[61]. Annavajhala *et al* tested the effectiveness of monoclonal antibodies against a pseudo-viral coronavirus model containing each of the mutations, including E484K and S477N. The results showed that the B.1.526 strain containing S477N mutation showed little to no antigenic impact and was fully neutralized. However, several antibodies were impaired and overall neutralization was decreased when targeting the E484K mutation[62]. This impairment of viral neutralization was also seen with convalescent serum (also known as "survivor's plasma" containing antibodies or special proteins generated by the body's immune system to the novel coronavirus infection) when tested against the

E484K strain. It has been found that the E484 mutation can be altered by a K, Q, or P amino acid change, all of which have been shown to decrease neutralization in serum by limiting the antibody binding to the RBD[63]. When tested against Moderna and Pfizer-BioNTech vaccinated sera, their efficacy was not affected by the S477N mutation, but did show a decrease in neutralization on the E484K mutation[61],

The B.1.351 strain (also known as Beta, or 501Y. V2) was first identified in South Africa in late 2020 and was the predominant strain in the region at that time (however the Delta strain has been seeing an increase in prevalence within that region and globally as of recently). This variant has multiple mutations in the S protein including K417N, E484K, and N501Y. As seen with the B.1.526 strain, having the E484K mutation allows for decreased neutralization, along with the N501Y mutation discussed in the U.K strain. Collectively, when all three mutations are present there is an increase in the infectivity of the virus, as studied in a pseudovirus model. Additionally, the B.1351 strain demonstrated a decrease in the neutralization of both serum convalescent sera and Pfizer-vaccinated sera[64]. Moderna vaccine showed a decrease in neutralization against but was still able to uphold immunity to the B.1.351 strain[65]. Two more recently published clinical trial studies evaluated the efficacy of two vaccines, ChAdOx1 nCoV-19 (a replication-deficient chimpanzee adenovirus vector vaccine) and NVX-CoV2373 (an adjuvanted, recombinant nano-particle vaccine), against the B.1.1351 variant. The ChAdOx1 nCoV-19 showed no efficacy against mild-to-moderate COVID-19 while NVX-CoV2373 had an efficacy of 49.4% against symptomatic COVID-19 caused by B.1.351 variant[66, 67].

The B.1.1.28 strain was originally identified in Rio de Janeiro, Brazil in February 2020. This strain contains the E484K mutation as mentioned in the B.1.526 strain[68]. Sapkal *et al* found that the Covaxin vaccine (developed in India) significantly increased the neutralization against this strain[69]. However, the Pfizer-BioNTech and Moderna vaccinated sera showed a decrease in neutralization of this strain[70]. The P1 strain (also known as Gamma, or 20J/501Y.V3) belongs to the B.1.1.28 strain lineage, initially found in travelers that came to Japan from Brazil, but has multiple mutations making it to be more severe (e.g, increased infectivity and decreased antibody neutralization). The P1 strain has three notable mutations within the RBD, K417T, E484K, and N501Y, similar to that of the B. 1.351 strain identified in South Africa[71]. A study tested responses of the P1 strain to various monoclonal antibodies and the results showed that (1) the Adagio antibodies were able to completely neutralize P1; (2) a significant decrease in neutralization with most of the antibodies; and (3) bamlanivimab had no neutralization ability[72]. Similar to B. 1.351, the Pfizer-BioNTech and Moderna vaccinated sera showed a decrease in neutralization of P1 strain[70].

The most recent variant of significance is the B. 1.617 strain found in India in late 2020. It has developed into three subvariants, with the main variant being B.1.617.2 (also known as Delta). The strain possesses common signature mutations consisting of D111D (synonymous substitution), G142D, L452R, E484Q, D614G, and P681R, in the S protein. Within the RBD, three concerning mutations include L452R and E484Q, along with P681R in the furin cleavage site[73]. These mutations could result in increased ACE2 binding as seen with other strains that contain the mutations, as well as potentially increase the rate of S1-S2 cleavage resulting in better transmissibility. A recent study illustrated that strains that contain L452R mutation can escape host immune response by eluding both HLA-restricted and humoral immune responses[74]. Another study found that the L452R mutations had a decreased response to vaccines (Pfizer-BioNTech and Moderna) compared to the original strain[75].

New mutations have been shown to affect the current therapeutics, indicating the current vaccines can become inefficient in given circumstances. Thus, despite the tremendous efforts being made in vaccine development and distribution, long-term management like new vaccine development and additional booster (e.g., yearly) shots may be necessary to eradicate the current pandemic.

### 4 ANTIVIRAL DRUGS

Vaccine development has been a major focus for the prevention of SARS-CoV-2 in a healthy population. Other therapeutic approaches including antiviral drugs have been utilized and researched to treat patients with COVID-19. Major antiviral drugs that have been tested for COVID-19 treatment include remdesivir, hydroxychloroquine, and lopinavir-ritonavir. However, hydroxychlorquine and lopinavir-ritonavir are not utilized as often due to their controversial effectiveness. The U.S. FDA initially approved for EUA of hydroxychloroquine and chloroquine for hospitalized patients on April 24, 2020, but revoked on June 15, 2020 due to cardiovascular complications. The clinical trial of lopinavir-ritonavir was revoked by WHO on July 4, 2020 due to lack of mortality reduction[76, 77]. There are also monoclonal antibodies which are being developed for the treatment. The major antiviral drugs are listed in table 3.

Remdesivir is an antiviral drug that acts as a nucleoside analog inhibiting the viral RdRp of coronaviruses like SARS-CoV-2[78]. On October 22, 2020, the U.S. FDA approved the antiviral drug remdesivir (brand name: Veklury) for use in adults

**Table 3 Antiviral drugs**

| Drug name | Other name(s) | Mechanism of action | Approval status in treating COVID-19 |
|---|---|---|---|
| Remdesivir | Veklury (trade name) | Inhibiting the viral RNA-dependent RNA polymerase (RdRp) | U.S. FDA approved for EUA in adults and pediatric patients with severe symptoms on October 20, 2020. |
| Plitidepsin | Aplidin | Targeting eEF1A to inhibit ribosomal activity of host cells, so virus can't replicate | Phase Ⅲ clinical trial, conducted by PharmaMar, Inc. |
| Zotatifin | | Similar to plitidepsin, but targeting eIF4A | Phase Ⅰ clinical trial, conducted by Effector Therapeutics, Inc. |
| Molnupiravir | MK-4482/ EIDD-2801 | Converted to EIDD-1931 which acts as a substrate for the viral RdRp, leading to lethal mutagenesis of the virus | Phase Ⅱ/Ⅲ clinical trials, conducted by Merck Sharp & Dohme Corp. |
| Bamlanivimab | LY-CoV555 | mAb that targets overlapping epitopes in the RBD of S protein, preventing viral entrance | U.S. FDA approved for EUA in November 2020, revoked as single drug use in April 2021, still in EUA in combination therapies |
| Etesevimab | LY-CoV016, also known as JS016 | mAb that targets overlapping epitopes in the RBD of S protein, preventing viral entrance | U.S. FDA approved for EUA for combinational therapy of bamlanivimab and etesevimab on February 9, 2021, for mild to moderate patients |
| REGN-COV2 | Casirivimab & imdevimab | mAb that targets non-overlapping epitopes in the RBD of the S protein, preventing viral entrance | U.S. FDA approved for EUA on November 21, 2020, to treat mild to moderate patients. |

and pediatric patients (12 years of age and older and weighing at least 40 kg) for the treatment of COVID-19 requiring hospitalization. It also remains authorized for emergency use for the treatment of suspected or laboratory-confirmed COVID-19 in hospitalized pediatric patients weighing 3.5 kg (about 7.7 pounds) to less than 40 kg or hospitalized pediatric patients less than 12 years of age weighing at least 3.5 kg. The clinical study showed that remdesivir was superior to placebo in shortening the time to recovery in adults who were hospitalized with COVID-19 and had evidence of lower respiratory tract infection[79].

As some data suggested the benefits of remdesivir are not sufficient to consistently treat severe cases, therefore, trials of some new antiviral drugs are in the pipeline of development, including plitidepsin, zotatafin, and molnupiravir.

Plitidepsin is an antiviral drug that targets eukaryotic translation elongation factor 1 alpha 1 (eEF1A), which encodes an isoform of the alpha subunit of the elongation factor-1 complex, responsible for the enzymatic delivery of aminoacyl tRNAs to the ribosome[80, 81]. The study conducted by White *et al* found that plitidepsin was more potent in inhibiting SARS-CoV-2 in human cells than remdesivir[80]. On March 5, 2021, the Spanish pharmaceutical company PharmaMar started a clinical phase Ⅲ trial for plitidepsin for individuals with moderate to severe symptoms. A similar drug called zotatifin interacts with eukaryotic initiation factor-4A (eIF4A), which is important for the binding of the mRNA to the 40S ribosomal subunit. Zotatifin will be in a phase I clinical trial for treating symptoms in moderate to severely ill patients starting May 2021 by Effector Therapeutics, Inc. (USA).

Molnupiravir (also known as EIDD-2801/MK-4482), a prodrug of the antiviral ribonucleoside analog β-d-N4-hydroxycytidine (EIDD-1931), has been used in the past for the treatment of RNA viruses including influenza and coronaviruses[81, 82]. After molnupiravir enters the cells, it is converted to EIDD-1931, an active oral bioavailable ribonucleoside analog which is incorporated into viral RNA and interrupts the process of viral replication[82]. The drug is currently being tested in phase Ⅱ clinical trial with COVID-19 patients by Merck Sharp & Dohme Corp.

Currently, there are over 50 different monoclonal antibodies (mAb) being developed and tested as therapeutics to treat SARS-CoV-2. The antibodies are generally designed to target the S protein of SARS-CoV-2, but other options are to target the microenvironment to regulate local immune responses to viral infection. Many mAbs are in clinical trials, such as lenzilumab (Humanigen Inc.) which is a mAb targeting the granulocyte-macrophage colony-stimulating factor (GM-CSF), and risankizumab (collaboration between Boehringer Ingelheim and AbbVie) which is a mAb that targets IL-23. In this paper, we will discuss three U.S. FDA approved antibodies including bamlanivimab and etesevimab (Eli Lily and Co.), and REGN-COV2 (Regeneron, Inc.). These three mAbs all target the S protein of SARS-CoV-2. In November 2020, the U.S. FDA approved EUA for bamlanivimab alone to be used for treatment for mild to severe cases of SARS-CoV-2. However, in April of 2021, the EUA was revoked against bamlanivimab if given as a single drug due to increased resistance, but it can still be used with other drugs under EUA. Etesevimab has similar mechanisms to bamlanivimab, where they both bind overlapping epitopes in the RBD of the S protein. The combined therapy of bamlanivimab and etesevimab was approved by the FDA for EUA on February 9, 2021 for patients presenting with moderate COVID-19 symptoms. In a randomized clinical trial with mild to moderate cases, bamlanivimab monotherapy showed no decrease in viral load, but when given in combination with etesevimab, there was a significant drop in viral load[83]. The other drug, REGN-COV2, is a mAb cocktail produced by Regeneron that contains two mAbs: casirivimab and imdevimab. It targets the non-overlapping epitopes in the RBD of the S protein, thus preventing viral entrance to host cells. The U.S. FDA approved the cocktail EUA to treat mild to moderate cases in adults and pediatric patients on November 21, 2020[84].

### 5 DISCUSSION

Despite the rapid development of vaccines and ongoing clinical trials for other therapeutics, the SARS-CoV-2 infection is still a large public health concern. Generally, patients who had previously been infected with SARS-CoV-2 will have antibodies against the virus 6 months after initial infection. However, given the emergence of new strains and the uncertainty of the long-term reinfection rates and complications of the disease, manufacturers such as Pfizer and Moderna suggest that vaccinated individuals may need an additional third dose, which will also possibly lead to yearly doses of COVID-19 vaccinations. As discussed earlier, many of the new strains demonstrate qualities that allow them to defend themselves against complete neutralization from convalescent or vaccination sera. This poses a long-term battle as the current vaccinations are constructed from the original genome of SARS-CoV-2. This can also be a potential cause of reinfection by different strains. A study by Li *et al* tested vaccinated sera in a rhesus macaques model against the D614G, B.1.1.7, and B.1351 strains[85]. The results showed normal neutralization of D614G and B.1.1.7 strains, but with a decrease in neutralization of the B.1351 despite being vaccinated.

Although vaccines have been available across the globe, the distribution of vaccines has been biased towards wealthier nations (with ~60% of countries distributing 75% of available vaccines in 2021), and some other countries only received a very limited number of vaccines (https://coronavirus.jhu.edu/vaccines/us-states, fig. 4)[86]. For example, as of June 21, 2021, Mali has only vaccinated 0.2% of its population (https://coronavirus.jhu.edu/region/mali or https://graphics.reuters.com/world-coronavirus-tracker-and-maps/vaccination-rollout-and-access/). To stop the spread of pandemic, assistance must be available to all countries. The COVID-19 Vaccines Global Access (COVAX), a worldwide initiative aimed at equitable access to COVID-19 vaccines directed by WHO and other organizations, coordinates international resources to enable low-to-middle-income countries equitable access to COVID-19 tests, therapies, and vaccines.

Multiple surveillance methods have been used to monitor the spread of SARS-CoV-2. The nasopharynx and nasal swabs PCR tests have been the dominant form of testing. However, this method of testing is highly reliable on clinician and patient sampling techniques. Other techniques that are currently being used to test and monitor spread include saliva PCR tests, serology tests, and wastewater surveillance. Newer forms of testing for SARS-CoV-2 are in the process of being developed, including utilizing CRISPR-Cas9 technology. The CRISPR-Cas9 system uses endonucleases to cut viral DNA or RNA and amplify the sequences to allow for rapid, accurate, and inexpensive detection of the virus. The newly discovered LEOPARD-CRISPR-Cas9

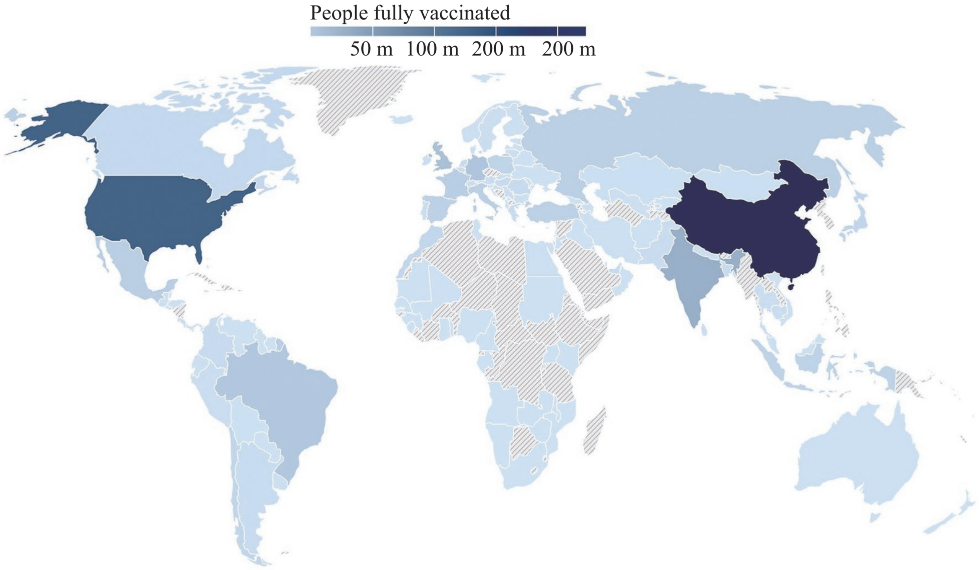

**Fig. 4** Vaccination progress across the world as of June 21, 2021 (https://coronavirus.jhu.edu/vaccines/us-states)
The map shows the COVID-19 vaccines are not equitably distributed around the world. Developed countries are vaccinating their populations faster than less developed countries. m: million technology can trace viral origin through the use of multiple guide RNAs simultaneously. This technology will potentially allow physicians to not only detect SARS-CoV-2 in patients, but also determine the specific strain(s) that cause the infection[87, 88].

The U.S. CDC provides timely updating information related to COVID-19 including travel guidance, variants, vaccines, and testing, which can be accessed at https://www.cdc.gov/coronavirus/2019-nCoV/index.html. Regardless of the vaccines or drugs that become available to combat COVID-19, it is still essential that individuals take the proper precautions and follow the guidelines set in place to prevent further spread. In conclusion, SARS-CoV-2 has caused millions of deaths worldwide since its origin in early 2020 and is still wreaking havoc. The development of vaccines against viruses has helped limit the spread but has yet to eradicate it. This is partially due to the rapid mutagenesis of the virus, resulting in dominant strains with qualities that allow an increase in infectivity, ability to escape host immune response, decreased neutralization from vaccination, and potentially demonstrate an increase in lethality. For the pandemic to be stopped, there are needs to be continuous development of new therapeutics as well as continuous monitoring and sequencing of the SARS-CoV-2 virus over time. There is also need to improve distribution and guidelines for receiving the vaccinations.

**Open Access**

This article is licensed under a Creative Commons Attribution 4.0 International License https://creativecommons.org/licenses/by/4.0/), which permits use, sharing, adaptation, distribution and reproduction in any medium or format, as long as you give appropriate credit to the original author(s) and the source, provide a link to the Creative Commons licence, and indicate if changes were made. The images or other third party material in this article are included in the article's Creative Commons licence, unless indicated otherwise in a credit line to the material. If material is not included in the article's Creative Commons licence and your intended use is not permitted by statutory regulation or exceeds the permitted use, you will need to obtain permission directly from the copyright holder. To view a copy of this licence, visit http://creativecommons.org/licenses/by/4.0/.

**Conflict of Interest Statement**

The authors whose names are listed in the manuscript certify that they have no affiliations with or involvement in any organization or entity with any financial interest (such as honoraria; educational grants; participation in speakers' bureaus; membership, employment, consultancies, stock ownership, or other equity interest; and expert testimony or patent-licensing arrangements), or non-financial interest (such as personal or professional relationships, affiliations, knowledge or beliefs) in the subject matter or materials discussed in this manuscript.
